# Anther culture in rice proportionally rescues microspores according to gametophytic gene effect and enhances genetic study of hybrid sterility

**DOI:** 10.1186/s13007-018-0370-z

**Published:** 2018-11-17

**Authors:** Yoshitaka Kanaoka, Daichi Kuniyoshi, Eri Inada, Yohei Koide, Yoshihiro Okamoto, Hideshi Yasui, Yuji Kishima

**Affiliations:** 1 Laboratory of Plant Breeding, Research Faculty of Agriculture, Hokkaido University, Sapporo, 060-8589 Japan; 2 Laboratory of Plant Breeding, Rakuno Gakuen University, Bunkyodai-Midorimachi, Ebetsu, 069-8501 Japan; 3 Plant Breeding Laboratory, Faculty of Agriculture, Kyushu University, 744 Motooka Nishi-ku, Fukuoka, Japan

**Keywords:** Anther culture, Callus, Hybrid sterility, Mapping, Microspore, *Oryza sativa*, *O. glaberrima*, Rice

## Abstract

**Background:**

To investigate plant hybrid sterility, we studied interspecific hybrids of two cultivated rice species, Asian rice (*Oryza sativa*) and African rice (*O. glaberrima*). Male gametes of these hybrids display complete sterility owing to a dozen of hybrid sterility loci, termed *HS* loci, but this complicated genetic system remains poorly understood.

**Results:**

Microspores from these interspecific hybrids form sterile pollen but are viable at the immature stage. Application of the anther culture (AC) method caused these immature microspores to induce callus. The segregation distortion of 11 among 13 known *HS* loci was assessed in the callus population. Using many individual calli, fine mapping of the *HS* loci was attempted based on heterozygotes produced from chromosome segment substitution lines (CSSLs). Transmission ratio distortion (TRD) from microspores was detected at 6 of 11 *HS* loci in the callus population. The fine mapping of *S*_*1*_ and *S*_*19*_ loci using CSSLs revealed precise distances of markers from the positions of *HS* loci exhibiting excessive TRD.

**Conclusions:**

We demonstrated that AC to generate callus populations derived from immature microspores is a useful methodology for genetic study. The callus population facilitated detection of TRD at multiple *HS* loci and dramatically shortened the process for mapping hybrid sterility genes.

**Electronic supplementary material:**

The online version of this article (10.1186/s13007-018-0370-z) contains supplementary material, which is available to authorized users.

## Background

Methods that maintain immature microspores as living cells can rescue pollen aborted between meiosis and fertilization and enable genetic studies of gametophytic characteristics. Anther culture (AC) is a technique in which plants are regenerated from microspores via callus formation in in vitro culture [1, 2]. Callus induced from microspores generally retains a haploid genome after meiosis in the parental plant, which can facilitate the characterization of genetic factors regulating the cell viability of male gametes. Here, we propose a methodology to apply this AC technique for the study of hybrid sterility (HS).

HS, which is a postzygotic isolation mechanism, typically refers to a deficiency in the reproductive capability of F_1_ hybrids [3]. In rice, HS is a well-known phenomenon that acts as a barrier to interspecific crossing, particularly between Asian rice (*Oryza sativa* L.) and African rice (*O. glaberrima* Steud.) [4, 5]. F_1_ hybrids between these two species produce almost no normal pollen, resulting in complete spikelet sterility. This pollen sterility is caused by the abortive effect of *HS* loci, generally known as *HS* loci, which reduce pollen and/or embryo-sac fertility only in heterozygotes [6–16]. Thirteen *HS* loci causing pollen sterility have been mapped thus far: gamete eliminator loci *S*_*1*_, *S*_*33*_*(t)*, and *S*_*37*_*(t)*, which cause abortion of both pollen grains and embryo sacs, and pollen killer loci *S*_*3*_, *S*_*18*_, *S*_*19*_, *S*_*20*_, *S*_*21*_, *S*_*29*_*(t)*, *S*_*34*_*(t)*, *S*_*36*_*(t)*, *S*_*38*_*(t),* and *S*_*39*_*(t),* which cause abortion of pollen grains (Table 1) [6–16]. Some of these *HS* loci induce allele-specific abortion owing to allelic conflicts at the *HS* locus in a gametophytic manner. For example, *sativa*–*glaberrima S*_*1*_ heterozygotes can only transmit the gamete carrying the *S*_*1*_^*g*^ allele from *O. glaberrima* to progenies, while the *S*_*1*_^*s*^ allele from *O. sativa* cannot participate in fertilization [17]. Such differential transmission between two alleles from their heterozygote is generally referred to as transmission ratio distortion (TRD) and results in segregation distortion (SD) in the progenies [17, 18]. Both male and female TRD by *S*_*1*_ is directly linked to the respective semi-sterility of pollen and embryo sacs in *S*_*1*_ heterozygotes. Major pollen sterility in *sativa*–*glaberrima* F_1_ hybrids results from the integration of multiple *HS* loci that individually may cause only partial sterility [18].

**Table 1:** Genotype segregation of markers linked to S loci in anther culture-induced calli derived from interspecific F1 hybrids between *O. sativa* and *O. glaberrima*

*S* loci	Function^a^	Chr.	Markers	No. of calli (frequency)^c^		*χ*^2^(1:1)	Allele transmitted preferentially via pollen^c^	References
				*s*	*g*			
*S*_1_	*Ge*	6	RM19359 RM204	5 (0.10)	47 (0.90)	33.92**	*g*	[6, 17, 34]
*S*_3_	*Pk*	11	RM536^b^	18 (0.35)	34 (0.65)	4.92*	g	[7]
*S*_18_	*Pk*	10	RM25321^b^	29 (0.56)	23 (0.44)	0.69	–	[8]
*S*_19_	*Pk*	3	RM60^b^	9 (0.17)	43 (0.83)	22.23**	*g*	[9, 35]
*S*_20_	*Pk*	7	RM82^b^	13 (0.25)	38 (0.75)	12.25 **	*g*	[8]
*S*_21_	*Pk*	7	RM429^b^	47 (0.90)	5 (0.10)	33.92**	*s*	[8]
*S*_29_(*t*)	*Pk*	2	RM279^b^	46 (0.88)	6 (0.12)	30.77**	–	[11]
*S*_34_(*t*)	*Pk*	3	RM7	26 (0.50)	26 (0.50)	0.00	–	[13]
*S*_36_(*t*)	*Pk*	2	RM207	29 (0.56)	23 (0.44)	0.69	–	[14]
*S*_37_(*t*)	*Ge*	1	RM449	33 (0.63)	19 (0.37)	3.77	*g*	[15, 37]
*S*_38_(*t*)	*Pk*	4	RM16260	27 (0.52)	25 (0.48)	0.08	–	[15]

Asterisks aberrant segregation to the theoretical ratio (s:g = 1:1): *p < 0.05; **p< 0.01. 1

^a^*Ge* gamete eliminator, *Pk* pollen killer

^b^unique markers selected based on the mapping in previous studies shown in Reference column

^c^s, O. *Sativa* allele; g, *O. glaberrima* allele; –, unknown

An ultimate goal of research on *HS* loci is the establishment of a strategy to overcome HS and to facilitate wide crossing for gene introgression or exploitation of heterosis. Two *S*_*1*_ locus components have recently been isolated in *sativa*–*glaberrima* interspecific hybrids [19, 20]. Dissection of *S*_*1*_ at the molecular level has taken over 20 years since its first discovery. A major rate-determining step in research on pollen killer and gamete eliminator genes in rice interspecific hybrids is successive backcrossing to dilute severe HS; this process, which requires the isolation of an *HS* locus from a number of *HS* loci, is a time-consuming facet of the genetic analysis [21, 22]. Rapid, efficient methods to identify functional *HS* loci and isolate additional loci in any cross combination are thus needed.

Here, we propose a new method for rapidly detecting *HS* loci using AC of interspecific hybrids. During callus induction from microspores, individual *HS* loci may exhibit differences in TRD leading to SD in the callus population, similar to observations in regenerated plants [23–25]. This phenomenon might be applicable to the mapping and identification of *HS* loci using AC calli derived from heterozygotes. Because successive backcrosses would not be required, such a method would drastically shorten the time required for detection and genetic analysis of *HS* loci.

In this study, we verified that the AC method successfully triggered callus formation in microspores of *sativa*–*glaberrima* interspecific F_1_ hybrids and then examined the segregation patterns of known *HS* loci. Our results allowed us to infer whether the lethal effect of each *HS* locus due to SD appeared at early microspore developmental stages in the callus population. Finally, we fine-mapped *S*_*1*_ and *S*_*19*_ loci using the anther-derived calli of their heterozygotes, which were produced from chromosome segment substitution lines (CSSLs) [26].

## Methods

### Plant materials

For the first experiment, we produced interspecific F_1_ hybrids by crossing *O. sativa* L. ssp. *japonica* with *O. glaberrima* Steud.. The donor parents, *O. glaberrima* accessions, Acc. IRGC 104038 from Senegal (designated as WK21) and Acc. IRGC 103777 from Mali (designated as WK18), were kindly provided by the International Rice Germplasm Center, the International Rice Research Institute, Philippines and had been conserved in Kyushu University. The *japonica* cultivar Nipponbare was reciprocally crossed with WK21 and WK18; the resulting F_1_ lines were named as N/WK21 and WK21/N in the first case and N/WK18 and WK18/N in the second (collectively referred to as WK/N).

The materials used in the second experiment were produced from two CSSLs, GIL27 and GIL31, which are introgression lines carrying segments from WK21 in the genetic background of *O. sativa* ssp. *japonica* ‘Taichung 65’ (T65) (Additional file 1: Fig. S1) [26]. GIL27 and GIL31 were obtained from a set of *O. glaberrima* introgression lines maintained by Kyushu University under the auspices of the National Bioresource Project. GIL31 and GIL27 carry chromosomal segments from *O. glaberrima* corresponding to the *HS* loci S_*1*_ and *S*_*19*_, respectively (Additional file 1: Fig. S1). To produce *S*_*1*_ and *S*_*19*_ heterozygotes, GIL31and GIL27 were backcrossed with T65, resulting in GIL31/T65 and GIL27/T65, respectively (collectively referred to as GIL/T65). All strains used in this study were grown according to conventional cultivation methods in a greenhouse at Hokkaido University. Starting at the seven-leaf stage, a short-day treatment (10 h light/14 h dark) was applied to induce reproductive growth.

### Anther culture

The two types of heterozygotes, WK/N and GIL/T65, and their parental lines were subjected to AC. Spikes enveloped by leaf sheaths were sampled at the booting stage. After excision of leaf blades and surface sterilization with 70% ethanol, the panicles were incubated at 10 °C (low temperature treatment) in the dark for 4–10 days. Approximately 70 anthers per dish were plated onto N6 [27] (Additional file 2: Table S1a), RI-13 [28] (Additional file 2: Table S1b), or SK-1 [29] (Additional file 2: Table S1c) callus-induction medium (CIM) prepared in a ø 90 mm × H 15 mm plastic dish. The plated anthers were hermetically sealed and then cultured at 25 °C in the dark for 4 months. Growing callus was transplanted to fresh medium to promote further growth.

### Classification of microspore developmental stages

**Fig. 1:**
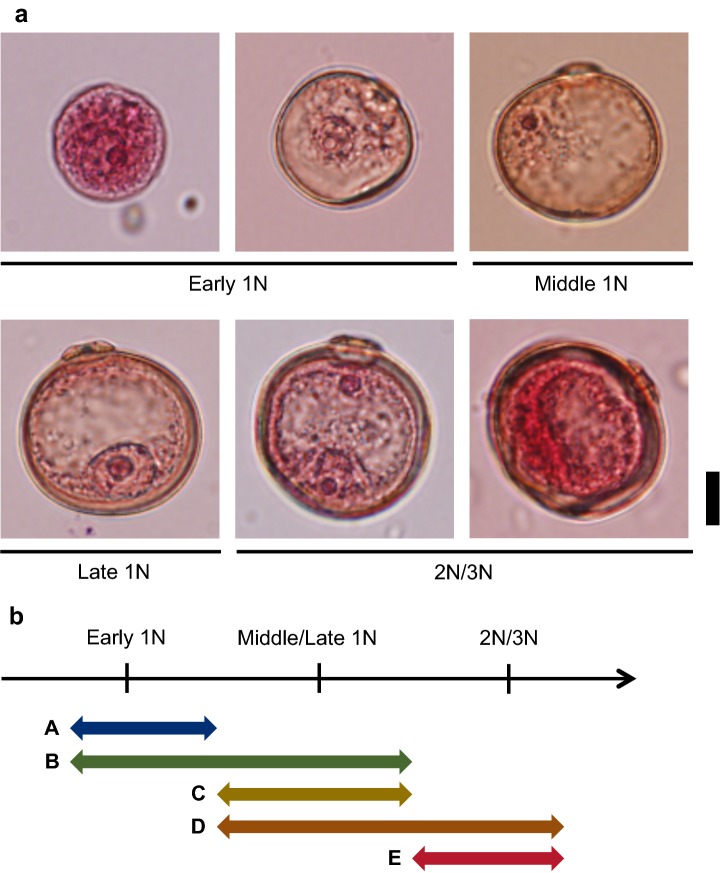
Classification of microspores based on developmental stages. Developmental stages of microspores were judged by microscopic observation after low temperature treatment, and the classes of anthers or calli were defined on the basis of these stages. **a** Microspores stained with acetocarmine at different developmental stages. Bar, 10 µm. **b** Categorization of microspore developmental stages into classes A to E. 1N, 2N, and 3N indicate uninucleate, binucleate, and trinucleate stages, respectively

To collect microspores at different developmental stages for use in the AC experiment, primary rachis-branches of each panicle were separated at the booting stage from top to bottom into three parts: first to third branches, fourth to sixth branches, and sixth to ninth branches, representing late, middle, and early stages, respectively. The youngest and oldest anthers in each group were sampled and fixed with formaldehyde–acetic acid–ethanol solution after 10 °C incubation. Microspores in the fixed spikelets were stained with acetocarmine, and their developmental stages were determined by optical microscopy as described previously [30]. Five microspore developmental stages, namely, classes A, B, C, D, and E, were defined as follows: A, early uninucleate (1N) and earlier stages; B, early 1N to middle/late 1N stages; C, the middle/late 1N stage; D, middle/late 1N to binucleate (2N) stages; and E, 2N and later stages (Fig. 1).

### Genotyping of AC-generated calli and detection of SD

Genomic DNA was extracted from AC-generated calli of WK/N and GIL/T65 and from leaves of F_2_ individuals of GIL/T65 following the method of Martin et al. [31] with one modification: calli were ground in elution buffer without freezing. A total of 104 calli induced from WK/N were used for the genotyping analysis. The genotyping was carried out with 11 *HS*-loci-linked simple sequence repeat (SSR) markers showing polymorphism between *O. sativa* and *O. glaberrima* according to previous studies (cited in Table 1 and Additional file 2: Table S2). PCR amplifications for genotyping were performed using GoTaq Green Master Mix (Promega), with the resulting products subjected to agarose gel electrophoresis. Other genotyping analyses of calli from GIL/T65 were focused on segregation of the SSR markers linked to *S*_*1*_ and *S*_*19*_ (Additional file 2: Table S2).

The homozygosity and heterozygosity of each callus was judged according to the SSR marker genotyping results. Calli retaining any heterozygous alleles were excluded from further analyses, as a callus with heterozygous alleles might not have been derived from a microspore with a haploid genome. Calli that were homozygous at all markers were subjected to the following genetic analyses. The SD of each marker was evaluated with a Chi square goodness-of-fit test based on a theoretical ratio of *s* (*ss*):*g* (*gg*) = 1:1 under no TRD. The segregation in two F_2_ populations derived from GIL/T65 plants was evaluated to confirm the gametophytic lethal effects of *S*_*1*_ and *S*_*19*_. Genomic DNAs isolated from F_2_ plants were genotyped with SSR markers adjacent to *S*_*1*_ and *S*_*19*_ loci (Additional file 2: Table S2). The genotype frequency of each marker was assessed by a Chi square test for goodness of fit to the Mendelian ratio (*ss*:*sg*:*gg *= 1:2:1). Another test was performed to distinguish the effect of the pollen killer from that of the gamete eliminator. In the case of the complete abortion of only male gametes possessing an *s* allele at a pollen killer locus, such as *S*_*19*_, the expected segregation of this locus would be *sg*:*gg *= 1:1. The segregation of the gamete eliminator locus *S*_*1*_ was expected to fit neither of these two ratios.

### Linkage mapping of S_1_ and S_19_ based on TRD

Genotype data of calli from GIL/T65 were used for linkage mapping of the partial lethal-factor locus causing TRD [32, 33]. The chromosomal positions of *S*_*1*_ and *S*_*19*_ were calculated according to Cheng et al. [32, 33]. Each *HS* locus was assumed to be adjacent to two SSR markers, one on each side. Recombination values between an *HS* locus and each of the two SSR markers were designated as *r*_*1*_ and *r*_*2*_, respectively. The parameter *t* (where 0 < *t* < 1) was used to denote the viability of the killed genotype (*sativa*-type alleles for *S*_*1*_ and *S*_*19*_) relative to the killer genotype (*glaberrima*-type alleles for *S*_*1*_ and *S*_*19*_). Recombination values on each interval of any two adjacent markers were used as initial values for *r*_*1*_ and *r*_*2*_ subject to repeated calculations, and parameter *t* was initialized to the frequency of non-recombinant *sativa*-type calli relative to non-recombinant *glaberrima*-type calli. Calculations were terminated when any of the parameters became zero or the relative errors of all parameters became less than 1 × 10^−6^. The *HS* locus was considered to be located in the interval in which *t* was minimized and neither *r*_*1*_ nor *r*_*2*_ were zero. The locations estimated for *S*_*1*_ and *S*_*19*_ were collated with the mapping results of previous studies [34, 35].

## Results

### Callus induction in interspecific F_1_ hybrids

We obtained microspore-derived calli from interspecific F_1_ hybrids whose male gametes were completely sterile because of the lethal effect of multiple *HS* loci. A total of 104 calli were obtained from 40,092 anthers of WK/Ns plants plated on CIM (Additional file 2: Table S3). In addition to N6 medium, we used RI-13 and SK-1 media as CIM, both of which have been reported to be suitable for callus formation in *O. glaberrima* and *O. sativa* ssp. *indica*, respectively, but not in *O. sativa* ssp. *japonica* cultivars [27–29]. Among the three AC media tested on F_1_ hybrid lines N/WK21 and WK21/N, RI-13 gave the highest callus formation rates (Additional file 2: Table S3). We therefore used RI-13 as the CIM for the remainder of the study. Of the three parental lines used as controls, the highest callus formation rate on RI-13 CIM was observed in the *japonica* cultivar Nipponbare (4.37%); the two *O. glaberrima* cultivars, WK21 and WK18, had rates of 1.10% and 2.12%, respectively. Callus formation rates of WK/Ns were lower than those of the parental lines.

We next determined the optimum microspore developmental stage for callus formation based on classes A to E (Fig. 1) in F_1_ (WK21/N) and parental lines. WK21/N and Nipponbare had the highest callus formation rates (0.93% and 5.87%, respectively) during the class C period (early 1N to middle/late 1N stages), and WK21 exhibited high rates during the class C period (1.99%) and also from middle/late 1N–2N stages (class D) (2.02%) (Additional file 2: Table S4). As reported for *japonica* rice cultivars [30], class C (i.e. middle and late 1N stages) was typically the most suitable period for efficient callus formation from anthers of *O. glaberrima* and its hybrid with *japonica*.

### Genetic constitutions of HS loci in interspecific hybrid calli

Eleven *HS* loci are currently known to be HS factors involved in pollen sterility in interspecific F_1_ hybrids between *O. sativa* and *O. glaberrima* [6–15] (Table 1). Heterozygotes of gamete eliminator loci *S*_*1*_ and *S*_*37*_*(t)* and pollen killer loci *S*_*3*_, *S*_*19*_, and *S*_*20*_ preferentially transmit *O. glaberrima* alleles (*g*) to progenies, whereas heterozygotes of pollen killer locus *S*_*21*_ transmit *O. sativa* alleles (*s*) [7, 16, 17, 36, 37].

We genotyped the 104 microspore-derived calli in this study using SSR markers closely linked to known *HS* loci. Among the 104 calli, 52 were found to be haploid or to be completely homozygous for alleles from either *O. sativa* or *O. glaberrima* according to all markers examined, which suggests that these calli were derived from normal microspores after meiosis (Additional file 1: Fig. S2). The remaining 52 calli possessed genomes containing a mixture of homozygous and heterozygous alleles at the examined loci. The incomplete genetic fixation in these calli may have been derived from microspores generated by abnormal meiotic processes, e.g., unreduced gamete formation. These calli were therefore excluded from further analysis of SD. We investigated the segregation of *HS* alleles of completely homozygous calli by genotyping SSR markers located near each *HS* locus. Significant SD was observed for more than half of the 11 genotyped *HS* loci, with the segregation of *S*_*1*_, *S*_*3*_, *S*_*19*_, *S*_*20*_, *S*_*21*_, and *S*_*29*_*(t)* found to be markedly distorted (Table 1). Because the segregation pattern of these *HS* loci was consistent with previous reports described above, the gametophytic effects of some *HS* loci involved in pollen viability determination were reflected in the genotype frequencies of calli derived from microspores of interspecific F_1_ plants. In contrast, no significant SD was detected for five *HS* loci, including *S*_*37*_*(t)*, and TRD at these loci should not have occurred at the 1–2N microspore developmental stages used for AC.

### SD of S_1_ and S_19_ caused by pollen semi-sterility

The SD of *HS* loci observed in calli from interspecific F_1_ hybrids resulted from allele-specific abortion of microspores. We therefore considered using genotype data from these calli for rapid mapping of *HS* loci without the need for multiple generations from crossing experiments. To confirm the feasibility of mapping *HS* loci using microspore-derived calli, we carried out this strategy with *S*_*1*_ and *S*_*19*_ loci. Because *S*_*1*_ is the major factor influencing HS between *O. sativa* and *O. glaberrima* and one of the most well-known gamete eliminators, detailed information on the location and function of this locus is available [19, 20, 34]. *S*_*19*_ is also one of the best-studied pollen killers, and its TRD characterization and fine mapping have been performed [35]. Heterozygotes subjected to AC were prepared from *O. glaberrima* introgression lines (GILs) in an *O. sativa* genomic background [26]. Although mainly composed of chromosomes from *O. sativa* T65, the genomes of GIL31 and GIL27 also contained some large pieces of chromosomal fragments from *O. glaberrima*. GIL31 and GIL27 were homozygous for *glaberrima* alleles at *S*_*1*_ and *S*_*19*_ located on chromosomes 6 and 3, respectively (Additional file 1: Fig. S1). To produce *S*_*1*_ and *S*_*19*_ heterozygotes, GIL31 and GIL27 were respectively crossed with T65, and the progenies were designated as GIL/T65.

**Fig. 2:**
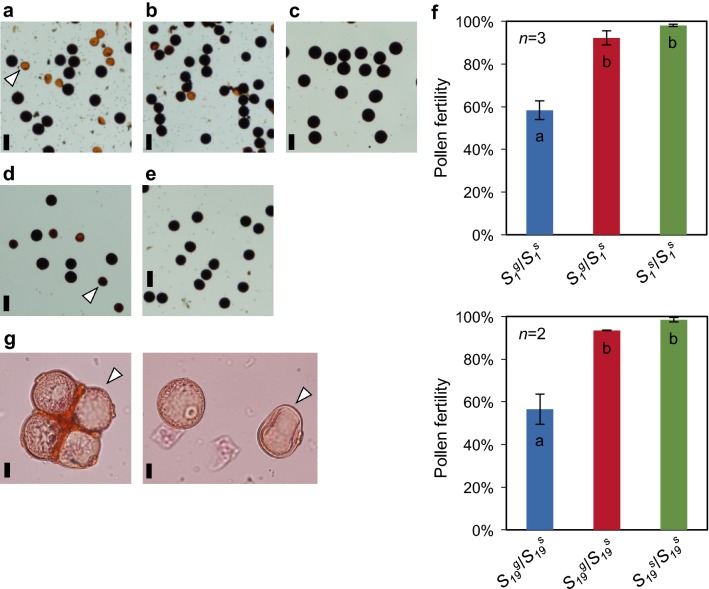
Microscopic observations of pollen semi-sterility caused by *S*_*1*_ and *S*_*19*_ in the heterozygous state. **a**–**e** Mature pollen stained with I_2_-KI solution in **a** *S*_*1*_^*g*^/*S*_*1*_^*s*^ heterozygotes, **b** *S*_*1*_^*g*^ homozygotes, **c** *O. sativa* ‘Taichung65’ (*S*_*1*_^*s*^ and *S*_*19*_^*s*^ homozygotes), **d** *S*_*19*_^*g*^/*S*_*19*_^*s*^ heterozygotes, and **e** *S*_*19*_^*g*^ homozygotes. Bar, 50 µm. **f** Pollen fertility exhibited by each genotype of *S*_*1*_ and *S*_*19*_ loci. Error bars indicate standard errors. Different lowercase letters between bars indicate significant differences at the 5% level (Welch’s *t* test corrected by Bonferroni’s method). **g** Aberrant microspores (indicated by the arrowhead) of *S*_*1*_^*g*^/*S*_*1*_^*s*^ heterozygotes stained with acetocarmine at the late uninucleate stage. Bar, 10 µm

To confirm the effect of *S*_*1*_ and *S*_*19*_ in GIL/T65, pollen fertility was examined. GIL31/T65 produced normal pollen grains at a rate of 58.3%, which compares with 92.2% and 98.0% for GIL31 and T65, respectively (Fig. 2a). Similarly, the mature pollen grains of GIL27/T65 were semi-sterile, whereas its parental lines were highly fertile. If two or more *HS* loci had independently disrupted microspore development, pollen fertility would have been theoretically reduced to less than 25%. Consequently, *HS* loci other than *S*_*1*_ and *S*_*19*_ did not appear to be present in GIL31/T65 and GIL27/T65 genomes, respectively. Abortive microspores in GIL31/T65 were readily observed after the late 1N stage and were characterized by weak acetocarmine staining, disappearance of contents of the microspores following the progression of developmental stages, and hollow grains at the mature pollen stage (Fig. 2). In contrast, abortive microspores in GIL27/T65 were not distinguishable from normal ones until the early 2N stage and seemed to accumulate less starch in the latter stage of pollen development (Fig. 2a). As far as the microscopic observation was concerned, the gametophytic lethal effect of *S*_*1*_ was shown in microspores during the class C, while the effect of *S*_*19*_ appeared on the later than the class C. Genotyping of F_2_ plants from GIL31/T65 with the SSR marker RM19359 at the *S*_*1*_ locus revealed an apparent distortion of the *S*_*1*_^*g*^ homozygous allele (54/56 plants) (Additional file 2: Table S5). As revealed by the F_2_ plants, the preferential transmission of the *S*_*1*_^*g*^ allele to the progenies through both male and female gametophytes validated an earlier finding that the *S*_*1*_^*g*^ allele is a gamete eliminator causing male and female TRD [6]. In contrast, *S*_*19*_^*g*^ homozygotes (20/42 plants) were excessively segregated in F_2_ plants derived from GIL27/T65, and the 1:1 segregation ratio for *gg*:*sg* genotypes at the *S*_*19*_ locus suggests that the *S*_*19*_^*g*^ allele functions as a pollen killer as reported previously (Additional file 2: Table S5) [9].

### AC-induced callus formation of GIL/T65 and distortions at S_1_ and S_19_ loci

In both GIL31/T65 and its parental lines, higher rates of AC-induced callus formation were observed in C- and D-class microspore stages compared with classes B and E (Additional file 2: Table S6). No callus was obtained from class A microspore. The maximum rate of callus formation in GIL31/T65 was 16.4%, which was less than half that of the parental lines. Rates were almost identical between the two parental lines. These results indicate that no locus existed that accounted for the different callus formation efficiencies between *s* and *g* alleles within the genomic region including the *S*_*1*_ locus. Consequently, the SD observed in this region in the callus population from GIL31/T65 can be considered to be due to TRD by the region including the *S*_*1*_ gene. A total of 1685 of the 2299 calli obtained in this study were randomly selected and genotyped with four SSR markers surrounding the *S*_*1*_ locus. Most of the genotyped calli possessed the *S*_*1*_^*g*^ allele, which corresponds to extreme SD towards the *S*_*1*_^*g*^ type (Table 2). The SD observed in the earlier microspore stage class B was comparable to that in classes C and D. The fates of microspores derived from the *S*_*1*_^*g*^/*S*_*1*_^*s*^ heterozygous parent were thus likely determined before the middle 1N stage in an allele-dependent manner.

**Table 2:** Genotype segregation of markers linked to the *S*1 locus in anther culture-induced calli derived from *S*1 heterozygotes

	No. of callus (frequency)^a^							
	RM7399		RM19359		RM204		RM276	
	*s*	*g*	*s*	*g*	*s*	*g*	*s*	*g*
*Class*								
B	1	24	0	25	1	24	1	24
	(0.04)	(0.96)	0.00	(1.00)	(0.04)	(0.96)	(0.04)	(0.96)
C	67	1108	4	1171	52	1166	270	905
	(0.06)	(0.94)	(0.00)	(1.00)	(0.05)	(0.96)	(0.23)	(0.77)
D	6	355	2	361	18	344	80	285
	(0.02)	(0.98)	(0.01)	(0.99)	(0.05)	(0.95)	(0.22)	(0.78)
E	2	13	2	13	2	13	5	10
	(0.13)	(0.87)	(0.13)	(0.87)	(0.13)	(0.87)	(0.33)	(0.67)
Total	76	1500	8	1570	73	1547	356	1224
	(0.05)	(0.95)	(0.01)	(1.00)	(0.05)	(0.96)	(0.23)	(0.78)

Class A to D are based on the developmental stage of microspores

^a^s: *O*. *sativa* allele; g: *O. glaberrima* allele

Calli from GIL27/T65 obtained from class-C microspores accounted for 21.1% of the total number of cultured anthers (3114; Additional file 2: Table S7). The similar rates of callus formation between the two parents confirms that the SD around the *S*_*19*_ locus was due simply to the gametophytic effect of the *S*_*19*_ gene. Distortion similar to that seen at the *S*_*1*_ locus was observed at the heterozygous *S*_*19*_ locus of AC-induced calli of GIL27/T65 (Table 3). The segregation of RM132 and RM14349, DNA markers adjacent to the *S*_*19*_ locus, was significantly distorted by calli carrying the *S*_*19*_^*g*^ allele. The different SDs of the SSR markers around the two *HS* loci may have resulted from each genetic recombination frequency. The detection of SD at the class-B microspore stage implies that the TRD associated with *S*_*19*_ occurred even earlier than the middle 1N microspore stage (Table 3).

**Table 3:** Genotype segregation of markers linked to the *S19* locus in anther culture-induced calli derived from *S19* heterozygotes

No. of callus (frequency)^a^				
	RM132		RM14349	
	*s*	*g*	*s*	*g*
*Class*				
B	4	24	5	24
	(0.14)	(0.86)	(0.17)	(0.83)
C	50	408	56	404
	(0.11)	(0.89)	(0.12)	(0.88)
D	4	20	0	23
	(0.17)	(0.83)	0.00	(1.00)
Total	58	452	61	451
	(0.11)	(0.89)	(0.12)	(0.88)

Class B to D are based on the developmental stage of microspores

^a^s, *O. sativa allele*; g, *O. glaberrima* allele

### Fine mapping of S_1_ and S_19_ using AC-induced callus genotypes

**Fig. 3:**
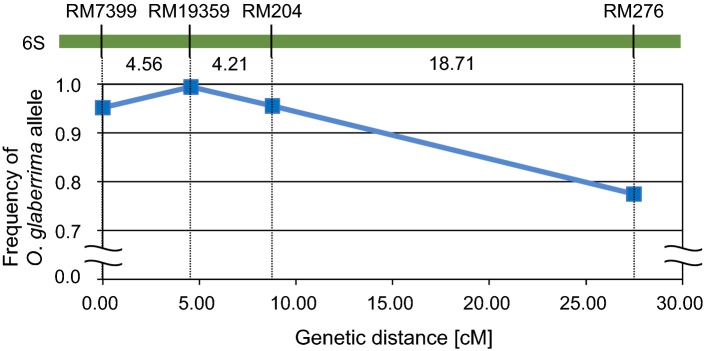
Unimodal plot of segregation distortion in the *S*_*1*_ region in anther culture (AC)-induced calli derived from *S*_*1*_ heterozygotes. The frequency of the *O. glaberrima* allele in AC-induced calli from *S*_*1*_ heterozygotes was detected with simple sequence repeat (SSR) markers (left axis). Genetic distances (below the green line) between SSR markers were calculated from recombination values using Kosambi’s mapping function

The degree of SD, which should be relaxed in proportion to the genetic distance of a marker from a TRD-causative gene, can be plotted on a unimodal chart [18, 32, 33]. The maximum SD at the *S*_*1*_ locus was observed at the SSR marker RM19359, with the SD of other markers reduced because they were distant from the causative gene (Fig. 3). This reduction in SD as a function of distance from the *S*_*1*_ locus can be explained by meiotic recombination. The differing degrees of SD, which are due to meiotic recombination and reflect the distance between a causative gene and a marker, can be effectively applied for fine mapping of *HS* loci in a callus population derived from their heterozygotes.

**Fig. 4:**
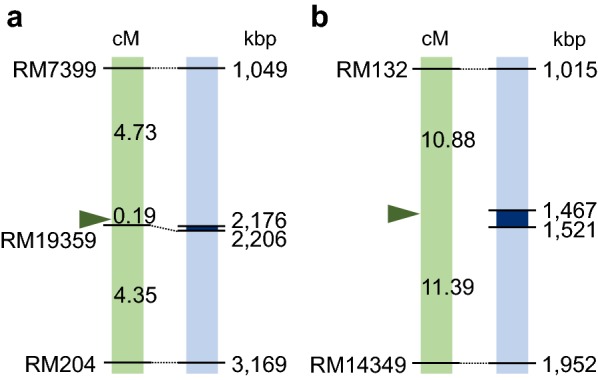
Linkage analyses of *S*_*1*_ and *S*_*19*_ based on the genotypes of anther culture-induced calli. The positions of *S*_*1*_ (**a**) and *S*_*19*_ (**b**) loci (green bars), determined according to likelihood estimates (Additional file 2: Table S8) based on TRD from microspore callus induction in this study, are compared with their known positions (blue bars) [34, 35]. Genetic distances were calculated from the recombination parameters using Haldane’s mapping function. Arrowheads indicate putative locations of *S*_*1*_ (**a**) and *S*_*19*_ (**b**). Blue bars represent the physical map of the corresponding region based on the *Oryza sativa japonica* ‘Nipponbare’ reference genome (IRGSP-1.0). The dark blue area on each blue bar represents the region in which *S*_*1*_ (**a**) or *S*_*19*_ (**b**) was mapped [34, 35, 42]

We mapped *S*_*1*_ and *S*_*19*_ using the genotype data obtained for AC-induced calli from GIL31/T65 and GIL27/T65, respectively, based on the method described by Cheng et al. [33]. The genotype data were analyzed without considering microspore developmental stages because the degree of SD was almost identical among the developmental classes (Tables 2 and 3). The two lethal factors, *S*_*1*_ and *S*_*19*_, were respectively located at their expected positions on chromosomes 6 and 3 (Fig. 4, Additional file 2: Table S8). The lethal factors for *S*_*1*_ on the short arm of chromosome 6 have recently been identified by map-based cloning [19, 20, 34]. Our linkage mapping using the AC-induced calli precisely located *S*_*1*_ between RM7399 and RM19359 on chromosome 6 (Fig. 4a). The probable location of the pollen killer *S*_*19*_ was mapped between RM132 and RM14349 on chromosome 3 (Fig. 4b), consistent with a previous study [35]. Both *S*_*1*_ and *S*_*19*_ loci caused extreme TRD through callus formation because of the low *t* values at the putative *HS* loci (0.33 ± 0.15% at *S*_*1*_ and 1.97 ± 0.63% at *S*_*19*_). Although SD of the two *S*_*19*_ markers, RM132 and RM14349, slightly recovered depending on their genetic distances from *S*_*19*_, the *t* estimate for *S*_*19*_ conformed to the severe SD that has been previously observed [35, 36]. These results demonstrate the applicability of using genotype data from AC-induced calli to construct fine maps for *HS* loci.

## Discussion

### Availability of AC-induced calli for the genetic study of HS

**Fig. 5:**
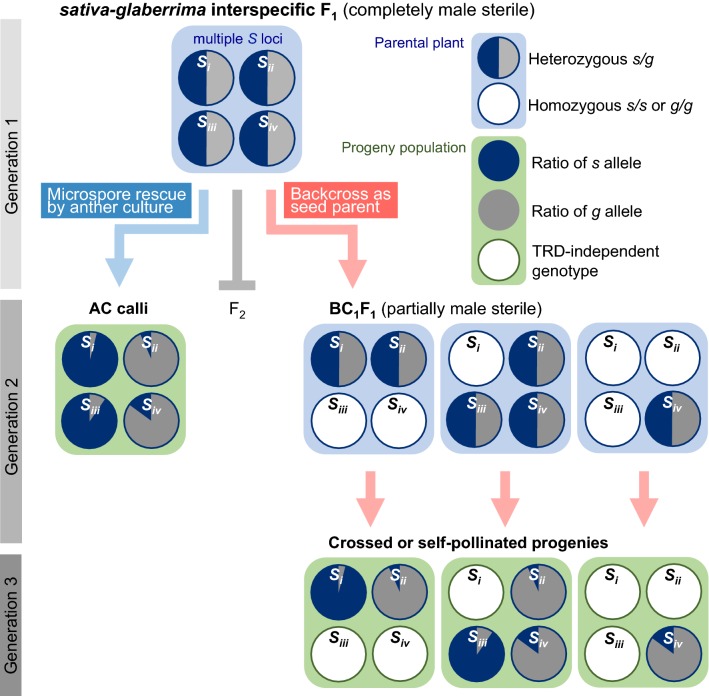
Schematic representation of rapid, multiple detection of male transmission ratio distortion (TRD) using anther culture (AC)-induced calli derived from interspecific F_1_ hybrids compared with the conventional method. The *sativa*–*glaberrima* (*s*–*g*) interspecific F_1_ population should possess four *HS* loci: *Sa*, *Sb*, *Sc*, and *Sd*. Blue boxes indicate parental plants displaying genotypes of each *HS* locus, and green boxes indicate progeny population of plants or calli displaying ratio of each genotypes resulted from TRD or either homozygous allele. Blue and red arrows indicate the flows to the conventional backcrossing method and the method using AC, respectively. With the conventional method, TRD for *HS* loci can appear at the earliest in the BC_1_F_1_ generation after backcrossing as the seed parent. F_2_ generation is unable to proceed owing to male gamete lethality. In AC-induced calli, which retain completely homozygous genotypes after meiosis of F_1_ plants, TRD at *HS* loci can be directly detected without backcrossing

In this study, we found that *HS* loci responsible for TRD of male gametophytes can act as segregation distorters in AC-induced calli (Table 1). This gametophytic effect was observed to directly influence the frequency of genotypes in AC-induced calli arising from *HS*-locus heterozygotes. This characteristic makes AC-induced callus a useful material to analyze TRD of male gametes involved in HS. Genetic analysis using an AC-induced callus population can accelerate the detection of *HS* loci and greatly reduce the amount of time required for mapping *HS* loci compared with the conventional backcrossing method (Fig. 5). When the conventional method is used, the F_1_ *sativa*–*glaberrima* offspring must be backcrossed as a female parent to either of the parental strains because of complete male sterility due to interactions at a dozen of *HS* loci. The male sterility-related phenotype caused by the *HS* locus appears at the end of reproductive organ development in BC_1_F_1_ plants, and TRD of male gametes can be detected no earlier than the next generation (Fig. 5).

In this study, we simultaneously detected SD of six *HS* loci, namely, *S*_*1*_, *S*_*3*_, *S*_*19*_, *S*_*20*_, *S*_*21*_, and *S*_*29*_*(t)*, in anther-cultured calli induced from microspores of WK/Ns, the hybrids between *O. sativa* and *O. glaberrima*. These instances of SD detected in the single AC callus population can be regarded as examples of TRD occurring during microspore development. Nevertheless, SD in a callus population might not always be linked to *HS* loci, but may sometimes be influenced by other factors, such as callus-formation efficiency. In previous studies using *japonica*–*indica* hybrids, distorted chromosomal regions were compared between F_2_ plants and AC doubled-haploid plants derived from the same F_1_ plants [23–25]. These comparisons revealed that the doubled-haploid plant population gave rise to different kinds of genomic distortions; some loci underwent SD in both the doubled-haploid plant population and F_2_ plants, whereas the SD of other loci was unique to doubled-haploid plants. These different cases of SD may have been due to various causes of TRD, such as gametophytic interactions in the hybrids, different efficiencies in the process of callus induction from microspores, or plant regeneration from callus. To confirm whether the TRD of *HS* loci was detectable in the interspecific F_1_ AC calli, further experiments were therefore performed using two CSSLs containing either *S*_*1*_ or *S*_*19*_ segments. The degree of SD uncovered by these experiments precisely reflected the distance from the position of each *HS* locus (Tables 2, 3; Fig. 3). The location of a potential *HS* locus can be inferred from loci exhibiting excessive TRD based on the segregation of a single marker or likelihood estimation using linked markers. In this study, the use of AC-induced calli from interspecific hybrids allowed us to estimate the location of an *HS* locus by analyzing the degree of TRD using DNA markers comprising a linkage group surrounding the locus.

HS is assumed to be due to the accumulated effects of multiple *HS* loci [5, 18]. In intraspecific hybrids, an *HS* locus that causes sterility is dependent on the cross combination and can be discovered from various crossing experiments [38, 39]. The conventional method used for detection and mapping of *HS* loci requires at least three generations to establish initial BC_1_F_2_ materials, with more laborious work needed thereafter to sort out each *HS* locus (Fig. 5). In contrast, the AC method allowed us to comprehensively screen and map loci causing TRD of male gametes in the interspecific F_1_ population just after the first hybridization. The genotype segregation of AC-induced calli can be effectively used for rapid detection of *HS* loci causing TRD. Moreover, this strategy can be applied to other crops if in vitro callus formation or embryogenesis from microspores is possible. However, the efficiency of callus induction is considered to be a limiting factor to evaluate TRD. The interspecific hybrids may cause low frequency of callus induction unable to appropriately map TRD factors.

### Relationship between SD and microspore development

AC-induced calli should lose their identity as microspores and possess characteristics similar to vegetative tissue (e.g. roots) [40, 41]. The deleterious effect of *HS* genes should appear only in reproductive tissue involved in gametogenesis. Consequently, *HS* genes are unlikely to function in microspores after the initiation of callus induction. Pollen semi-sterility caused by gametophytic lethal effects of *S*_*1*_ and *S*_*19*_ have been previously reported to occur at the 2N stage or even later [17, 35]. In the present study, our microscopic observations suggested that microspore abortion caused by *S*_*1*_ and *S*_*19*_ occurred after the late 1N and 2N stages, respectively, whereas the pattern of SD due to *S*_*1*_ and *S*_*19*_ in AC-induced calli from GIL/T65 provided evidence for the occurrence of allele-specific abortion prior to the 1N stage (Tables 2 and 3). Our results using the AC method suggest that gametophytic lethal effects of *S*_*1*_ and *S*_*19*_ genes arose during early microspore development. In contrast to six *HS* loci, namely, *S*_*1*_, *S*_*3*_, *S*_*19*_, *S*_*20*_, *S*_*21*_, and *S*_*29*_*(t)*, that displayed SD in AC-induced calli, five *HS* loci, namely, *S*_*18*_, *S*_*34*_*(t)*, *S*_*36*_*(t)*, *S*_*37*_*(t)*, and *S*_*38*_*(t)*, underwent no significant SD in AC-induced calli from WK/Ns (Table 1). Several different reasons can be invoked to explain why these five *HS* loci did not exhibit SD in AC-induced calli: (1) allele-specific abortion may have taken place after the 2N stage of microspore development; (2) no SD occurred, but pollen sterility was caused by a sporophytic effect; or (3) the *HS*-locus antagonistic relationships detected in *sativa*-*glaberrima* hybrids did not occur between Nipponbare and WK21.

## Conclusions

Taken together, our results demonstrate that the genotype segregation patterns of *HS* loci in AC-induced calli associated with microspore developmental stages are informative for understanding when *HS* loci determine the viability of male gametes. In this study, we have provided the first evidence that *S*_*1*_ and *S*_*19*_ genes initiate gametophytic pollen abortion before the middle 1N stage. Observations based on callus induction of microspores should not only accelerate genetic analysis of *HS* loci but also deepen our understanding of their functional characteristics.

## Additional files

**Additional file 1: Figure S1.** Chromosomal positions of *O. glaberrima* fragments in GIL31 (a) and GIL27 (b). Boxes represent chromosomal segments of GIL31 and GIL27 based on genotype data obtained from Oryzabase (https://shigen.nig.ac.jp/rice/oryzabase/). The different colored regions indicate homozygous Taichung65 fragments (white), homozygous IRGC104038 fragments (blue), and heterozygous or undetermined fragments (gray). The black bars on the right sides of boxes indicate homozygous IRGC104038 fragments on chromosome 6 of GIL31 (a) and chromosome 3 of GIL27 (b) harboring *S*_*1*_ and *S*_*19*_, respectively. ** Figure S2.** Graphical representation of genotypes of calli derived from interspecific F_1_ hybrids between *O. sativa* and *O. glaberrima*. A total of 104 anther culture-induced calli were evaluated using 11 simple sequence repeat (SSR) markers linked to *HS* loci; 52 calli were completely homozygous at all markers, and 52 exhibited partial heterozygosity. Cell colors indicate allele genotypes at the 11 SSR loci as follows: yellow, homozygous for the *glaberrima* allele; green, homozygous for the *sativa* allele; red, heterozygous.

**Additional file 2: Tabe S1.** Composition of callus induction media. (a) N6; (b) RI-13; (c) SK-1. **Tabe S2.** SSR markers for genotyping of *HS* loci. **Tabe S3.** Summary of the results of callus induction by anther culture of interspecific F_1_ hybrids between *O. sativa* and *O. glaberrima.* **Tabe S4.** Callus formation rates from anther culture of interspecific F_1_ hybrids between *O. sativa* and *O. glaberrima.* **Tabe S5.** Segregation distortion in self-pollinated progenies of *S*_*1*_ and *S*_*19*_ heterozygotes. **Tabe S6.** Callus formation rates from anther culture of *S*_*1*_ heterozygotes. **Tabe S7.** Callus formation rates from anther culture of *S*_*19*_ heterozygotes. **Tabe S8.** Maximum likelihood estimates of recombination rates and viability parameters.

## Abbreviations

AC: anther culture (AC)
HS: hybrid sterility (HS)
CSSLs: chromosome segment substitution lines (CSSLs)
TRD: transmission ratio distortion (TRD)
SD: segregation distortion (SD)
N/WK21: F_1_ line between Nipponbare (seed parent) and WK21 (pollen parent)
WK21/N: F_1_ line between WK21 (seed parent) and Nipponbare (pollen parent)
N/W18: F_1_ line between Nipponbare (seed parent) and WK18 (pollen parent)
WK18/N: F_1_ line between WK18 (seed parent) and Nipponbare (pollen parent)
WK/N: collective name for N/WK21, WK21/N, N/WK18, WK18/N
T65: Taichung 65
GILs: *O. glaberrima* introgression lines
GIL31/T65: GIL31 backcrossed with T65 (and, respectively) and
GIL27/T65: GIL27 backcrossed with T65
GIL/T65: collective name for GIL31/T65 and GIL27/T65
CIM: callus-induction medium
SSR: simple sequence repeat
