# Supplementary material for: Anther culture in rice proportionally rescues microspores according to gametophytic gene effect and enhances genetic study of hybrid sterility
Source: Plant Methods. 2018 Nov 17;14:102. doi: 10.1186/s13007-018-0370-z (PMC6240274; doi:10.1186/s13007-018-0370-z)
Supplement: Supplementary file 1 — Additional file 1: Figure S1. Chromosomal positions of O. glaberrima fragments in GIL31 (a) and GIL27 (b). Boxes represent chromosomal segments of GIL31 and GIL27 based on genotype data obtained from Oryzabase (https://shigen.nig.ac.jp/rice/oryzabase/). The different colored regions indicate homozygous Taichung65 fragments (white), homozygous IRGC104038 fragments (blue), and heterozygous or undetermined fragments (gray). The black bars on the right sides of boxes indicate homozygous IRGC104038 fragments on chromosome 6 of GIL31 (a) and chromosome 3 of GIL27 (b) harboring S1 and S19, respectively. Figure S2. Graphical representation of genotypes of calli derived from interspecific F1 hybrids between O. sativa and O. glaberrima. A total of 104 anther culture-induced calli were evaluated using 11 simple sequence repeat (SSR) markers linked to HS loci; 52 calli were completely homozygous at all markers, and 52 exhibited partial heterozygosity. Cell colors indicate allele genotypes at the 11 SSR loci as follows: yellow, homozygous for the glaberrima allele; green, homozygous for the sativa allele; red, heterozygous. [file 13007_2018_370_MOESM1_ESM.pdf]

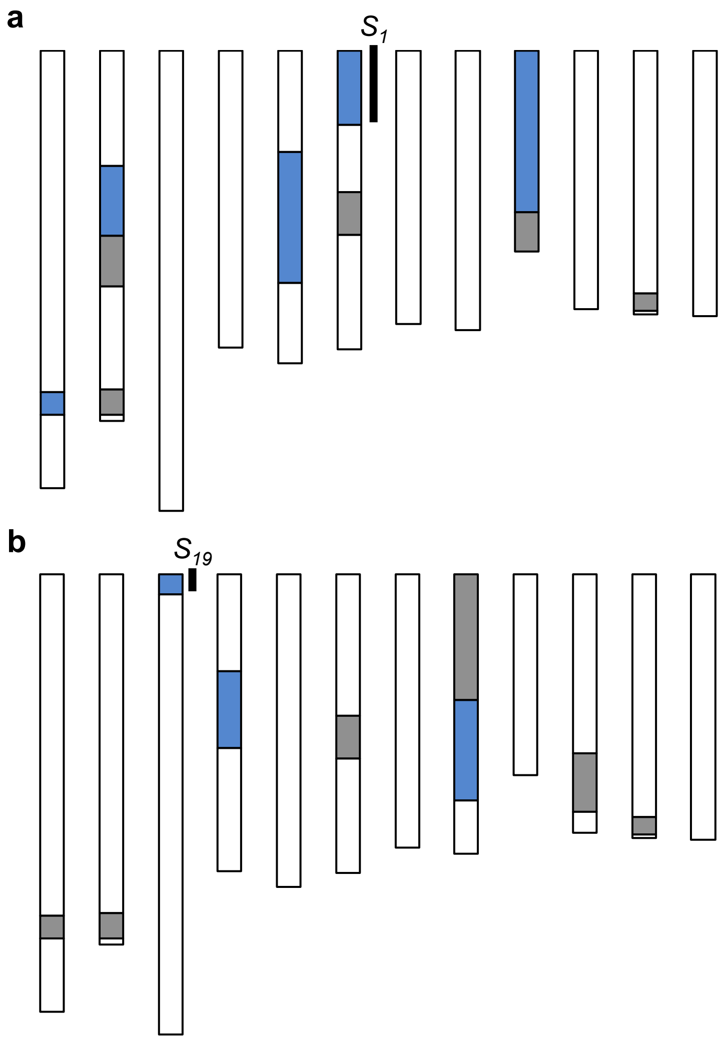

Supplemental figure 1. The chromosomal positions of *O. glaberrima* fragments in GIL31 (a) and GIL27 (b).
Each box represents the chromosomal segments of GIL31 and GIL27, based on the genotype data obtained from Oryzabase (https://shigen.nig.ac.jp/rice/oryzabase/). White, homozygous Taichung65 fragments; blue, homozygous IRGC104038 fragments; gray, heterozygous or undetermined fragments. Bars on the right side of the box indicate the homozygous IRGC104038 fragments on chromosome 6 of GIL31 (a) and chromosome 3 of GIL27 (b), which contain S_1 and S_{19}, respectively.

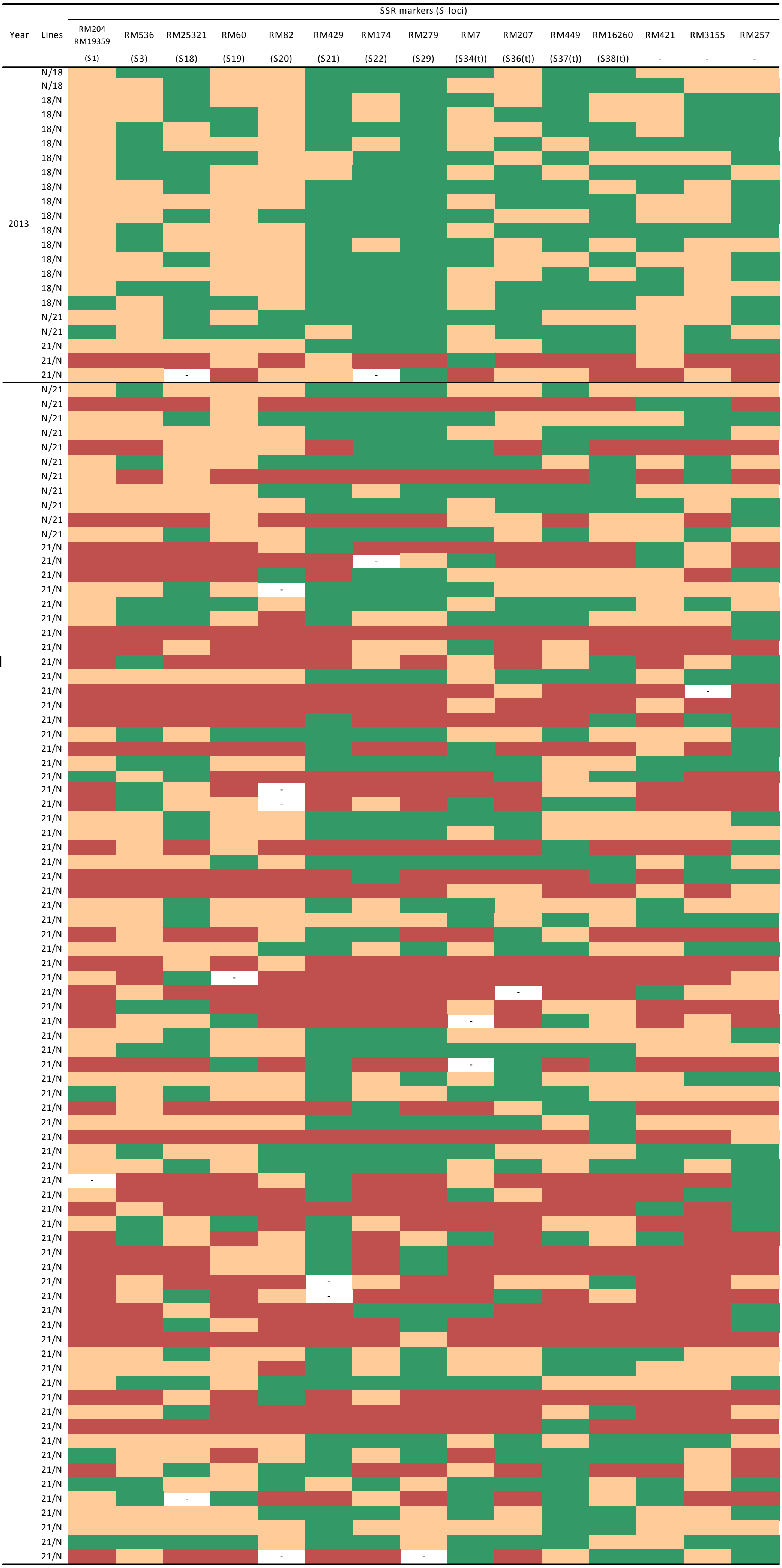

Supplemental figure 2. Graphical genotypes of calli derived from interspecific F_1 hybrids between *O. sativa* and *O. glaberrima*.

A total of 104 AC calli were evaluated with 11 SSR markers linked *S* loci, and 52 showed complete homozygosity at all the markers, while the other 52 calli showed partial heterozygosity. Cell color indicates alleles at 11 SSR loci as *yellow* for homozygous of *glaberrima allele*, *green* for *sativa* homozygous allele, *red* for heterozygous.
