# Supplementary material for: Anther culture in rice proportionally rescues microspores according to gametophytic gene effect and enhances genetic study of hybrid sterility
Source: Plant Methods. 2018 Nov 17;14:102. doi: 10.1186/s13007-018-0370-z (PMC6240274; doi:10.1186/s13007-018-0370-z)
Supplement: Supplementary file 2 — Additional file 2: Tabe S1. Composition of callus induction media. (a) N6; (b) RI-13; (c) SK-1. Tabe S2. SSR markers for genotyping of HS loci. Tabe S3. Summary of the results of callus induction by anther culture of interspecific F1 hybrids between O. sativa and O. glaberrima. Tabe S4. Callus formation rates from anther culture of interspecific F1 hybrids between O. sativa and O. glaberrima. Tabe S5. Segregation distortion in self-pollinated progenies of S1 and S19 heterozygotes. Tabe S6. Callus formation rates from anther culture of S1 heterozygotes. Tabe S7. Callus formation rates from anther culture of S19 heterozygotes. Tabe S8. Maximum likelihood estimates of recombination rates and viability parameters. [file 13007_2018_370_MOESM2_ESM.pdf]

Table S1a Composition of N6 callus induction medium

Materials		Final (/L)
Chu (N6) Medium Salt Mixture (Wako)	KNO_3	2830 mg
	$(NH_4)_2SO_4$	463 mg
	KH_2PO_4	400 mg
	$MgSO_4 \cdot 7H_2O$	185 mg
	$CaCl_2 \cdot 2H_2O$	166 mg
	KI	0.8 mg
	H_3BO_3	1.6 mg
	$MnSO_4 \cdot 4H_2O$	4.4 mg
	$ZnSO_4 \cdot 7H_2O$	1.5 mg
	$Na_2 \cdot EDTA$	37.25 mg
	$FeSO_4 \cdot 7H_2O$	27.85 mg
Vitamines	Glycine	2 mg
	Thiamine HCl	1 mg
	Pyridoxine HCl	0.5 mg
	Nicotinic acid	0.5 mg
Hormones	2,4-D	0.5 mg
	NAA	2.5 mg
	Kinetin	0.5 mg
	Sucrose	50 g
	Aspartic acid	1 g
	Glutamine	1 g
	Gellangum	2 g

Table S1b Composition of RI-13 callus induction medium

Materials		Final (/L)
Solution 1	KNO_3	2275 mg
	NH_4NO_3	600 mg
	NaH_2PO_4・2H2O	172 mg
	MgSO4・7H2O	247 mg
	CaCl2	222 mg
Solution 2	KI	0.75 mg
	H3BO3	3 mg
	MnSO4・4H2O	13.195 mg
	ZnSO4・7H2O	2 mg
	Na2MoO4・2H2O	0.25 mg
	CuSO4・5H2O	0.025 mg
	CoCl2・6H2O	0.025 mg
Solution 3	Na2EDTA	37.25 mg
	FeSO4・7H2O	27.85 mg
Solution 4	Thiamine HCl	10 mg
	Pyridoxine HCl	1 mg
	Nicotinic acid	1 mg
Hormones	2,4-D	2 mg
	NAA	2.5 mg
	Kinetin	0.5 mg
	Sucrose	50 g
	Aspartic acid	1 g
	Glutamine	1 g
	Gellangum	2 g

Table S1c Composition of SK-1 callus induction medium

Materials		Final (/L)
Solution 1	KNO3	3150 mg
	(NH4)2SO4	231.245 mg
	KH2PO4	540 mg
	MgSO4・7H2O	185 mg
	CaCl2	113.265 mg
Solution 2	KI	1 mg
	H3BO3	6 mg
	MnSO4・4H2O	22.3 mg
	ZnSO4・7H2O	1.5 mg
	Na2MoO4・2H2O	0.25 mg
	CuSO4・5H2O	0.025 mg
Solution 3	Na2EDTA	37.25 mg
	FeSO4・7H2O	27.85 mg
Solution 4	Glycine	2 mg
	Thiamine HCl	2.5 mg
	Pyridoxine HCl	2.5 mg
	Nicotinic acid	2.5 mg
Hormones	2,4-D	0.5 mg
	NAA	2.5 mg
	Kinetin	0.5 mg
	Sucrose	50 g
	Aspartic acid	1 g
	Glutamine	1 g
	Gellangum	2 g

Table S2 SSR markers for genotyping of *S* loci

S loci	Chr.	Markers	Position [Mbp]		Primer sequences	Annealing [°C]
S_1	6	RM7399	1.05	F	AATCTCTCTGCTGAGCCAGC	56
				R	TCTTGCCCTTGCAGCTAATC	
S_1	6	RM19359	2.21	F	GATCCAAGTAGGCACGAGTCACC	56
				R	AGCTGATTCCTCTCTGCTTCTCG	
S_1	6	RM204	3.17	F	GTGACTGACTTGGTCATAGGG	56
				R	GCTAGCCATGCTCTCGTACC	
S_1	6	RM276	6.23	F	CTCAACGTTGACACCTCGTG	56
				R	TCCTCCATCGAGCAGTATCA	
S_3	11	RM536	8.99	F	TCTCTCCTCTTGTTTGGCTC	56
				R	ACACACCAACACGACCACAC	
S_{18}	10	RM25321	12.81	F	CACTTCTTGTCACTGTACACACTCC	56
				R	CACATGCCGTATATCAACTACCG	
S_{19}	3	RM60	0.11	F	AGTCCCATGTTCCACTTCCG	56
				R	ATGGCTACTGCCTGTACTAC	
S_{19}	3	RM132	1.02	F	ATCTTGTTGTTTCGGCGGCGGC	66
				R	CATGGCGAGAATGCCCACGTCC	
S_{19}	3	RM14349	1.95	F	GATCCTTAGGCATGGAATGATGG	60
				R	CGTGTTCACTGAATATGGGAAAGC	
S_{20}	7	RM82	3.13	F	TGCTTCTTGTCAATTCGCC	56
				R	CGACTCGTGGAGGTACGG	
S_{21}	7	RM429	26.81	F	TCCCTCCAGCAATGTCTTTC	56
				R	CCTTCATCTTGCTTTCCACC	
$S_{29}(t)$	2	RM279	2.88	F	GCGGGAGAGGGATCTCCT	56
				R	GGCTAGGAGTTAACCTCGCG	
$S_{34}(t)$	3	RM7	9.83	F	TTCGCCATGAAGTCTCTCG	56
				R	CCTCCCATCATTTCGTTGTT	
$S_{36}(t)$	2	RM207	35.38	F	TCTGTCGGCAGACTCCTCTT	56
				R	CCACTTCCTCCATTGTGCTT	
$S_{37}(t)$	1	RM449	15.12	F	TTGGGAGGTGTTGATAAGGC	56
				R	ACCACCAGCGTCTCTCTCTC	
$S_{38}(t)$	4	RM16260	0.18	F	TGCTAGTAGGAGAGGAGCGAAGC	56
				R	ACTGTGAGTCGTGTCAGAGTTGG	

Table S3 Summary of the results of callus induction by anther culture of interspecific F1 hybrids between *O. sativa* and *O. glaberrima*

Lines	Medium	No. of anthers	No. of calli	Rate of callus formation [%]
N/WK18	N6	475	2	0.42
	SK-1	501	0	0
WK18/N	N6	1152	6	0.52
	SK-1	1197	9	0.75
N/WK21	RI-13	14724	11	0.07
	N6	2088	1	0.05
	SK-1	2116	1	0.05
WK21/N	RI-13	13457	71	0.53
	N6	2203	2	0.09
	SK-1	2179	1	0.05
Nipponbare	RI-13	2565	112	4.37
WK18	RI-13	992	21	2.12
WK21	RI-13	13239	146	1.1
	N6	1469	12	0.82
	SK-1	1484	8	0.54

Table S4 Callus formation rates from anther culture of interspecific F1 hybrids between *O. sativa* and *O. glaberrima*

Lines	Rate of callus formation [%]<br>No. of calli / No. of cultured anthers				
	A	B	C	D	E
WK21/N	0	0.44	0.93	0.57	0.3
	0 / 203	9 / 2048	32 / 3433	14 / 2450	16 / 5287
Nipponbare	-	4.67	5.87	1.63	0
	-	23 / 493	83 / 1415	5 / 306	0 / 264
WK21	0	0.23	1.99	2.02	0.32
	0 / 208	5 / 2187	51 / 2558	72 / 3556	14 / 4405

Class A to E are based on the developmental stage of microspores (see Figure 1).
-, no data

Table S5 Segregation distortion in self-pollinated progenies of *S1* and *S19* heterozygotes

Lines		F_2 (GIL31/T65)	F_2 (GIL27/T65)	
Genotype of parents		S_1^g/S_1^s	S_{19}^g/S_{19}^s	
Markers		RM19359	RM132	RM14349
No. of plants[1]	*gg*	54	24	26
	ss	0	2	1
	gs	2	16	15
	Total	56	42	42
χ^2(*gg*:*ss*:*gs*=1:1:2)		152.43 **	25.43 **	33.19 **
χ^2(*gg*:*gs*=1:1)		48.29 **	1.60	2.95

Asterisks indicate the aberrant segregation to the theoretical ratio: *, $p < 0.05$; **, $p < 0.01$.
1: s, *O. sativa* allele; g, *O. glaberrima* allele.

Table S6 Callus formation rates from anther culture of *S1* heterozygotes

Lines	Genotype	Callus-formation rates [%]<br>No. of calli / No. of cultured anthers			
		B	C	D	E
GIL31/T65	S_1^g/S_1^s	1.2	16.4	12.8	1.9
		39 / 3298	1743 / 10653	502 / 3192	15 / 810
T65	S_1^s/S_1^s	-	36.3	37.2	-
		-	465 / 1932	78 / 222	-
GIL31	S_1^g/S_1^g	-	24.1	35.1	-
		-	892 / 2460	136 / 366	-

Class B to D are based on the developmental stage of microspores (see Figure 1).
-, no data.

Table S7 Callus formation rates from anther culture of *S19* heterozygotes

Lines	Genotype	Callus-formation rates [%]<br>No. of calli / No. of cultured anthers		
		B	C	D
GIL27/T65	S_{19}^{g}/S_{19}^{s}	2.4	21.1	11.7
		11 / 468	656 / 3114	31 / 264
T65	S_{19}^{s}/S_{19}^{s}	-	31.5	-
		-	193 / 612	-
GIL27	S_{19}^{g}/S_{19}^{g}	-	32.4	-
		-	183 / 564	-

Class B to D are based on the developmental stage of microspores. -, no data.

Tble S8 Maximum likelihood estimates of recombination rates and viability parameters

Markers	Chr.	Position [kbp]	Parameters (±SE)		
			r_1	r_2	t
RM7399	6	1,049			
			0.0451 (±0.0053)	0.0019 (±0.0011)	0.0033 (±0.0015)
RM19359	6	2,206			
			0.0000 (±0.0000)	0.0417 (±0.0051)	0.0052 (±0.0018)
RM204	6	3,169			
			0.0000 (±0.0000)	0.1795 (±0.0097)	0.0491 (±0.0059)
RM276	6	6,231			
RM132	3	1,015			
			0.0978 (±0.0131)	0.1018 (±0.0134)	0.0197 (±0.0063)
RM14349	3	1,952			

The recombination and viability parameters were estimated for all the intervals composed of two adjacent SSR markers used for genotyping of anther-culture calli from S1 and S19 heterozygotes according to Cheng et al. (1998): the two estimates, r1 and r2, are the recombination values of the lethal-factor locus to the upper and lower SSR marker shown in the table, respectively; the estimate t is the relative viability, particularly the callus-induction efficiency in this study, of the sativa-type microspores to the gleberrima-type. The physical positions of the SSR markers are based on the O. sativa japonica cv. Nipponbare reference genome (IRGSP-1.0).
